# Assessment of ovarian reserve using anti-Müllerian hormone levels in benign gynecologic conditions and surgical interventions: a systematic narrative review

**DOI:** 10.1186/1477-7827-12-125

**Published:** 2014-12-15

**Authors:** Akira Iwase, Tomoko Nakamura, Tatsuo Nakahara, Maki Goto, Fumitaka Kikkawa

**Affiliations:** Department of Obstetrics and Gynecology, Nagoya University Graduate School of Medicine, 65 Tsurumai-cho, Showa-ku, Nagoya, 466-8550 Japan; Department of Maternal and Perinatal Medicine, Nagoya University Hospital, 65 Tsurumai-cho, Showa-ku, Nagoya, 466-8550 Japan

**Keywords:** AMH, Cystectomy, Endometriosis, Ovarian reserve, UAE

## Abstract

The usefulness of anti-Müllerian hormone (AMH) for the quantitative evaluation of ovarian reserve has been established. Therefore, serum AMH has been recently applied to the assessment of ovarian reserve outside infertility treatment. We conducted a computer-based search, using keywords, through the PubMed database from inception until May 2014 and summarized available studies evaluating ovarian damage caused by gynecologic diseases, such as endometriosis and ovarian tumor, as well as surgical interventions, such as cystectomy and uterine artery embolization (UAE), to discuss the usefulness of serum AMH. Most of the studies demonstrated a decline of serum AMH levels after cystectomy for endometriomas. It is not conclusive whether electrocoagulation or suturing is preferable. The effects of other gynecologic diseases and interventions, such as hysterectomy and UAE, on ovarian reserve are controversial. Serum AMH levels should be considered in determining the indication and selection of operative methods for benign gynecologic conditions.

## Background

Benign gynecologic diseases are often implicated in fertility problems, and therefore, fertility-preserving interventions are required for such conditions. However, surgical interventions involving the uterus and ovaries have been demonstrated to possibly affect ovarian function, as these interventions might decrease ovarian tissue levels and blood supply to ovaries [1–3].

The potential ovarian function at a given point in time is now defined as the “ovarian reserve,” which reflects the quality and quantity of follicles in the ovaries [4]. Therefore, interventions for gynecologic conditions aiming to preserve fertility as well as benign gynecologic diseases themselves must be assessed from the point of view of ovarian reserve.

Follicle-stimulating hormone (FSH), a widely used marker, was found to be insufficient for predicting ovarian reserve after in vitro fertilization (IVF) [5]. Among newly developed ovarian reserve tests, the serum level of anti-Müllerian hormone (AMH) has been recognized as an improved and informative marker [6, 7]. AMH is produced by granulosa cells from preantral and small antral follicles, and therefore, AMH levels indirectly represent the total number of follicles, as estimated by the number of early-growing-stage follicles [8, 9]. AMH is reported to be a better marker than FSH and inhibin B, and it displays similar performance as the antral follicle count in predicting ovarian response [10]. One of the advantages of serum AMH is its lower inter-cycle variability, distinguishing this variable from other markers including FSH and inhibin B [11], although serum AMH levels are reported to vary slightly during the menstrual cycle [12]. Therefore, many researchers have begun using serum AMH levels to evaluate ovarian damage caused by surgical interventions, such as ovarian cystectomy and uterine artery embolization (UAE). Moreover, serum AMH levels from conception to menopause in healthy females were analyzed to assess the value of AMH in predicting reproductive lifespan [13].

In the current review, we mainly focused on the assessment of ovarian reserve after gynecologic interventions. We also included an assessment of the influence of gynecologic diseases, such as endometriosis and endometriomas, on ovarian reserve to discuss what mechanisms are associated with the disturbance of ovarian reserve. Other ovarian toxic interventions, such as chemotherapy and radiotherapy, were not included in the current review to maintain a focus on primary gynecologic conditions.

## Methods

### Sources

Articles were identified through a PubMed database search covering the period from database’s inception through May 2014. We conducted a computer-based search of all articles published in English using the keywords “AMH/anti-Müllerian hormone and ovarian reserve” and [“endometrioma,” “endometriosis,” “ovarian tumor,” “ovarian cystectomy,” “uterine artery embolization/UAE,” “salpingectomy,” or “hysterectomy”].

### Study selection

We examined the 77 published studies identified using the aforementioned keywords. For inclusion, articles needed to describe the evaluation of ovarian reserve using serum AMH levels in relation to benign gynecologic conditions and/or interventions for such conditions. Abstracts and review articles were excluded. Articles evaluating ovarian reserve using other markers, such as inhibin B and the antral follicle count, were not included. Finally, 40 articles were chosen (22 for endometriomas/endometriosis, 6 for other ovarian tumors/cysts, and 12 for other interventions). In the current review, we adopted the unit used in the original studies to display AMH levels. Values presented in pmol/L can be converted to ng/mL by dividing them by 7.14.

## Results

### Ovarian tumor, surgery, and intervention

Surgical interventions involving the uterine adnexa can possibly cause damage to ovarian function because of the loss of the ovarian cortex and/or detrimental effects on blood supply to the ovaries [14]. Surgical procedures include cystectomy for endometriomas and other benign ovarian tumors, salpingectomy for hydrosalpinx, and UAE for uterine leiomyoma, all of which are often performed as part of infertility treatments. Therefore, declines of ovarian reserve after these interventions must be avoided as much as possible. Assessments of their influence on ovarian reserve using serum AMH levels may be helpful for improving these surgical interventions. On the contrary, ovarian cysts/tumors themselves might affect ovarian reserve. Several published reports have investigated this point.

### Endometrioma, endometriosis and ovarian reserve

The ovarian cortex surrounding endometriomas has been revealed to possibly cause a reduction in the volume of healthy tissue, a lower follicular density, and a loss of cortex-specific stroma [15–17]. These results suggest that endometriomas might result in diminished ovarian reserve. Lemos et al. first reported that infertile patients with minimal/mild endometriosis, categorized as revised American Society for Reproductive Medicine (rASRM) classification stage I/II [18], have decreased serum AMH levels compared to those in a control group who had tubal obstruction without endometriosis (1.26 ± 0.7 ng/mL in the study group [median age, 29.5 years] vs. 2.02 ± 0.72 ng/mL in the control group [median age, 30.5 years], mean ± SD; P = 0.004) [19]. It is not likely that the patients recruited in this study had endometriomas because all patients had rASRM stage I/II endometriosis. Concerning endometriomas, Kim et al. demonstrated that patients with stage IV endometriosis with endometriomas displayed significantly lower AMH levels than age- and BMI-matched controls (2.1 ± 0.3 ng/mL vs. 3.1 ± 0.4 ng/mL, mean ± SEM; P = 0.02) [20]. Similarly, it has been reported that AMH levels are lower in patients with stage III/IV endometriosis without prior ovarian surgery than in controls (0.97 ± 0.59 ng/mL in the study group [mean age, 33.6 ± 1.9 years] vs. 1.72 ± 0.63 ng/mL in the control group [mean age, 32.6 ± 2.0 years], mean ± SD; P = 0.001) [21]. Uncu et al. also reported lower AMH levels in patients with endometrioma than in age-matched controls (endometrioma vs. controls: 2.81 ± 2.15 ng/mL vs. 4.20 ± 2.26 ng/mL, mean ± SD; P = 0.002) [22]. In this study, the unilateral endometrioma group exhibited lower AMH levels than the bilateral endometrioma group. However, a cross-sectional study including 313 women with a diagnosis of endometriosis and 413 women without endometriosis (control) identified no difference in serum AMH levels related to the type of endometriosis excluding women who had a past history of surgery for endometriomas (4.1 ± 3.4 ng/mL for the control group, 4.5 ± 3.6 ng/mL for the superficial peritoneal lesion group, 3.8 ± 2.9 ng/mL for the endometrioma group, 3.4 ± 3.0 ng/mL for the deep infiltrating endometriosis group, mean ± SD; P = 0.06) [23].

### Surgery for endometrioma

Unlike the controversy regarding the influence of an endometrioma itself on ovarian reserve, it appears conclusive that cystectomy for endometriomas diminishes ovarian reserve. Chang et al. and Iwase et al. first reported that cystectomy for endometriomas leads to a greater decrease of serum AMH levels than cystectomy for other benign ovarian tumors [24, 25]. Subsequently, several similar studies have been published. Among these, only 1 study, in which preoperative serum AMH levels were lower for patients’ ages than those in the other studies, demonstrated that cystectomy for endometriomas did not cause significant changes in serum AMH levels [26, 27]. These studies have already been reviewed and meta-analyzed, which led to the conclusion that cystectomy for endometriomas may cause a decline of ovarian reserve [28, 29]. In addition to the studies comparing preoperative and postoperative AMH levels, a cross-sectional study also demonstrated that AMH levels were significantly lower in women who previously underwent endometrioma surgery, irrespective of whether endometriomas were present at the time of the study (P < 0.05) [23].

Measurements of serum AMH levels make the quantitative evaluation of the effects of surgery on ovarian reserve possible. Consequently, several issues have emerged regarding surgery for endometrioma and ovarian reserve. The first issue is the incidence of post-surgical decreases in ovarian reserve. Laparoscopic cystectomy for bilateral endometriomas has been reported to possibly cause a greater decline of serum AMH levels than unilateral cystectomy [24, 25]. Hirokawa et al. reported that the serum AMH levels 1 month after surgery compared with the preoperative levels were 24.7 ± 32.5 and 62.8 ± 29.6% lower for patients with unilateral and bilateral tumors, respectively (P < 0.001) [30]. They also found that severe endometriosis with higher rASRM scores tends to cause greater declines of serum AMH levels after surgery. Kitajima et al. reported that the decline of serum AMH levels within 3 months after surgery was significantly greater in women with an excised cyst wall that contained normal ovarian tissue than in women without healthy ovarian tissue in the excised cyst wall (42.0 ± 32.9% vs. 8.9 ± 13.4%; P = 0.01) [31]. In a 1-year follow up study, the immediate postsurgical decline of serum AMH levels was revealed to be possibly related to the reduction of cortex volume from the excision, and the medium/long-term decrease was believed to be caused by other factors, including reduced blood supply to the ovaries. [32]. Recently, a study of 193 patients who underwent laparoscopic cystectomy for endometrioma demonstrated that postoperative AMH levels significantly decreased after surgery irrespective of age (≤38 years, P < 0.001; >38 years, P < 0.001), cyst size (>3 cm, P = 0.018; ≤3 cm, P = 0.022), and laterality (unilateral, P < 0.001; bilateral, P < 0.001) [33].

Another interesting issue is how various surgical methods affect postoperative AMH levels. Cauterization and vaporization of the cyst wall might have an advantage in sparing ovarian reserve. The 3-step technique, consisting of irrigation in the first laparoscopy, followed by gonadotropin-releasing hormone agonists and vaporization in the second laparoscopy, resulted in a lower postsurgical decline of AMH levels than endometrioma stripping (3.9 ± 0.4 and 2.9 ± 0.2 ng/mL with stripping vs. 4.5 ± 0.4 and 3.99 ± 0.6 ng/mL with the 3-step technique, mean ± SEM at baseline and 6 months after surgery; P = 0.026 in the stripping group) [34]. Bipolar electrocoagulation might damage the cortex of ovaries. Ferrero et al. performed a randomized controlled trial (RCT) to compare serum AMH levels following hemostasis by bipolar coagulation versus suturing after laparoscopic cystectomy for bilateral endometriomas [35]. They found no significant difference in the mean percentage decrease of AMH levels in the 2 groups after 3, 6, and 12 months of follow-up. Another RCT was performed to compare hemostatic matrix with bipolar electrocoagulation after laparoscopic cystectomy for unilateral endometrioma [36]. Serum AMH levels in the bipolar coagulation group were significantly lower at 1 month after surgery and similar at 3 months after surgery compared to those in the hemostatic matrix group (hemostatic matrix vs. bipolar coagulation: 3.73 ± 1.50 ng/mL vs. 3.66 ± 1.20 ng/mL preoperative, 2.72 ± 1.49 ng/mL vs. 1.64 ± 0.93 ng/mL in the 1st month, and 3.07 ± 1.43 ng/mL vs. 2.84 ± 1.12 ng/mL in the 3rd month, mean ± SD; P = 0.001 and P = 0.467 in the 1st and 3rd months, respectively). Zaitoun et al. prospectively compared laparoscopic cystectomy with bipolar coagulation and laparotomic cystectomy with sutures. Only the laparoscopic cystectomy group displayed a significant decrease of AMH levels (4.5 ± 0.8, 2.4 ± 0.5, 2.7 ± 0.5, and 2.5 ± 0.4 ng/mL in the laparoscopic group vs. 4.6 ± 0.9, 4.5 ± 0.9, 4.4 ± 0.9, and 4.5 ± 0.9 ng/mL in the laparotomy group preoperatively and 6, 12, and 18 months after surgery, respectively; mean ± SD, P = 0.8, P < 0.005, P < 0.005, and P < 0.005 preoperatively and 6, 12, and 18 months after surgery, respectively) [37].

Another issue is the recovery of serum AMH levels after surgery for endometriomas. AMH is produced by primary, preantral, and small antral follicles but not primordial follicles [9]. If rearrangements of follicle cohorts from a healthy primordial follicle pool occur, then serum AMH levels that decreased as a result of surgery could recover. Two early studies indicated that serum AMH levels might be restored to some extent by 1 week, 1 month, and 3 months after cystectomy for endometriomas [24, 38]. In the aforementioned RCT, AMH levels, especially in the bipolar coagulation group, tended to recover at 3 months after surgery compared with 1 month after surgery [36]. On the contrary, several studies demonstrated that serum AMH levels gradually decreased or remained depressed [22, 35, 39, 40]. Celik et al. assessed serum AMH levels preoperatively and at 6 weeks and 6 months after surgery and reported that AMH levels gradually decreased patients with bilateral tumors or those with cyst diameters ≥5 cm [39]. Sugita et al. reported that serum AMH levels could be higher or lower 12 months after surgery compared to those 1 month after surgery [32].

### Other benign ovarian cysts

The effects of cystectomy for benign ovarian tumors on AMH levels have been assessed in a few studies. Iwase et al. reported that laparoscopic cystectomy for non-endometriomas (15 cases of unilateral tumors and 5 cases of bilateral tumors) caused a lesser, but significant, decline of AMH levels (3.92 and 3.29 ng/mL before and after cystectomy, respectively, median; P = 0.044) [25]. Similarly, another study reported that serum AMH levels were lower 1 week after laparoscopic cystectomy for non-endometriotic cysts (69.2% of the preoperative AMH levels; P < 0.05) [24]. Jang et al. recently reconfirmed the results from Iwase and Chang [41]. Thus far, only 1 prospective randomized study has compared serum AMH levels after cystectomy for unilateral non-endometriotic cysts using laparoscopy and laparotomy [42]. Significant differences were found in AMH levels between the laparoscopy and laparotomy groups at 1, 3, and 6 cycles after cystectomy (4.2, 3.2, 2.6, and 2.4 ng/mL in the laparoscopy group vs. 4.6, 3.7, 3.5, and 3.6 ng/mL in the laparotomy group before and 1, 3, and 6 cycles after cystectomy, respectively, mean; P = 0.180, P = 0.004, P < 0.001, and P < 0.001, respectively). No significant difference was observed in preoperative serum AMH levels between the mature cystic teratoma group and age- and BMI-matched controls (4.0 ± 0.5 ng/mL vs. 4.0 ± 0.5 ng/mL, mean ± SEM; not significant) [20]. The decline rates of serum AMH levels after cystectomy for benign ovarian tumors have been reported to be lower than those observed after cystectomy for endometriomas. Chang et al. reported that the decline in serum AMH levels at 1 week after laparoscopic cystectomy was smaller for non-endometriotic cysts than for endometrioma (69.2% vs. 33.9% of preoperative AMH levels; P = 0.028) [24]. A similar tendency was reported by Iwase et al. (the extent of decline compared to preoperative levels: 16.1% in the benign ovarian tumor group vs. 24.9% in the endometrioma group) [25]. On the contrary, it was recently reported that the rate of AMH decline at 3 months after laparoscopic cystectomy did not differ between the endometrioma group and the other benign ovarian cyst group (36.64 ± 29.20% vs. 30.58 ± 29.66% of the preoperative level; P = 0.36) [43].

### Other interventions that affect ovarian reserve

Hydrosalpinx can reduce the likelihood of pregnancy after IVF. Therefore, salpingectomy of diseased fallopian tubes before IVF should be considered [44]. Salpingectomy might result in decreased blood supply to the ovaries. However, the ovarian response after salpingectomy has been inconsistent [45, 46]. In a cross-sectional study, no significant differences were found in serum AMH levels among patients with varying tubal status, including 26, 34, 23, and 51 patients who underwent bilateral salpingectomy, unilateral salpingectomy, bilateral interruption in the proximal oviducts, and bilateral oviduct obstruction, respectively [47]. On the contrary, Grynnerup et al. reported that AMH levels were significantly lower in the salpingectomy group than in the non-salpingectomy group (16.1 pmol/L [median age, 34 years] vs. 23.4 pmol/L [median age, 33 years], median; P = 0.04) [48]. Sequential assessments of serum AMH levels did not reveal statistically significant differences before and after laparoscopic coagulation and dissection of the proximal tubes (1.548 ng/mL preoperatively vs. 1.481 ng/mL 3 months after surgery, median; P = 0.079) [49]. Similarly, research illustrated that serum AMH levels were not affected by tubal ligation (1.43 ng/mL preoperatively vs. 1.30 ng/mL 12 months after surgery, median; P = 0.23) [50].

Hysterectomy, even if the ovaries are preserved, has been reported to possibly cause adverse effects on ovarian function, which might shorten the time to menopause [51, 52]. Atabekoglu et al. reported that serum AMH levels tended to decline more at 4 months after hysterectomy compared to those in controls who did not undergo hysterectomy (1.46 ± 2.02 to 0.62 ± 0.9 ng/mL in the hysterectomy group vs. 1.53 ± 1.82 to 1.26 ± 1.78 ng/mL in the controls, mean ± SD, P = 0.73 and P = 0.262 before and after surgery, respectively) [53]. The authors concluded that the decrease in ovarian reserve after hysterectomy is possibly caused by hypoxia as a result of the interruption of the uterine arteries. However, Lee at al. revealed that hysterectomy did not affect ovarian artery blood flow or serum AMH levels (1.80 ± 1.81 ng/mL preoperatively in women aged 44.2 ± 3.5 years vs. 1.52 ± 1.72 ng/ml at 3 months after hysterectomy, mean ± SD; P = 0.805) [54]. Wang et al. demonstrated that serum AMH levels significantly declined 3 months following hysterectomy, whereas those 3 months after myomectomy were similar to the preoperative levels (1.08 ± 0.77 ng/mL vs. 0.81 ± 0.55 ng/mL in the hysterectomy group; P < 0.01; 1.54 ± 0.95 ng/mL vs. 1.50 ± 0.58 ng/mL in the myomectomy group; P = 0.07; mean ± SD preoperatively and 3 months after surgery) [55]. In another point of view, prophylactic salpingectomy with total laparoscopic hysterectomy (TLH) did not cause a significant decrease of AMH levels compared to those observed after standard TLH with adnexal observation (−0.06 ± 0.1 ng/mL vs. −0.08 ± 0.1 ng/mL ΔAMH for TLH with vs. without salpingectomy, mean ± SD; P = 0.35) [56]. This result was reconfirmed in an RCT (2.26 ± 2.72 ng/mL and 1.86 ± 1.99 ng/mL in the bilateral salpingectomy with hysterectomy group vs. 2.25 ± 2.57 and 1.82 ± 3.12 ng/mL in the no salpingectomy group, mean ± SD at baseline and 3 months after surgery; P = 0.99 and P = 0.97, respectively) [57].

**Figure 1:**
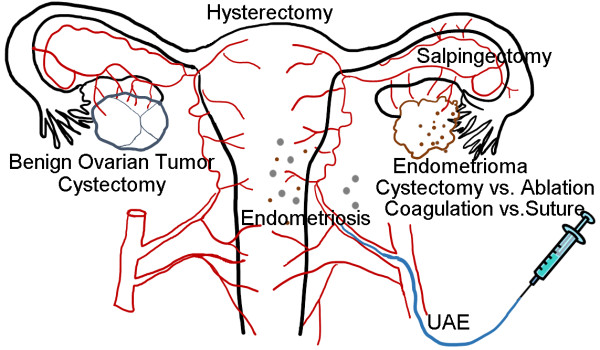
**Possible factors that affect ovarian reserve in patients with benign gynecologic conditions.** UAE, uterine artery embolization.

UAE has been introduced as an effective treatment for severe hemorrhage related to pregnancy and has been proposed as a replacement for hysterectomy for some patients with uterine leiomyoma [58, 59]. However, the possible decline of ovarian reserve after UAE remains controversial. Hehenkamp et al. measured serum AMH levels at baseline and at several time points during follow-up after UAE and hysterectomy and found that AMH levels remained significantly lower during the follow-up period in the UAE group but not in the hysterectomy group [60]. Similar results were reported by Arthur et al., who compared UAE and laparoscopic myomectomy (2.17 ng/mL after laparoscopic myomectomy vs. 0.78 ng/mL after UAE, median; P = 0.01) [61]. On the contrary, the combination of UAE and local methotrexate for interstitial pregnancies did not appear to reduce ovarian reserve based on AMH levels, although only 3 cases were analyzed [62]. Figure 1 presents the gynecologic diseases and interventions in which assessments of ovarian reserve using AMH have been reported.

## Discussion

### Main findings and limitations

As we reviewed manuscripts for this article, the following results were demonstrated: 1) the influence of endometriosis/endometrioma itself on ovarian reserve remains controversial, 2) cystectomy for endometriomas tends to reduce AMH levels more severely than that for other types of benign ovarian tumors, especially for bilateral or severe disease, 3) cystectomy and bipolar coagulation in endometrioma surgery could be the factors that decrease ovarian reserve. However, it is not conclusive which surgical method is preferable from the point of view of ovarian reserve, 4) although hysterectomy, salpingectomy, and UAE might affect ovarian reserve, there is insufficient evidence to draw a conclusion.

One of the limitations of our study is the high heterogeneity of the included articles. Serum AMH levels were primarily affected by the recruited patients’ ages, which differed among the studies. Another limitation is that the significance of the decrease in serum AMH levels after the interventions is not conclusive, as the changes were possibly encompassed by the wide normal range for serum AMH [13]. However, this review involved the systematic collection of articles to assess ovarian reserve in relation to gynecologic diseases and interventions and the narrative evaluation of the articles.

### Interpretation and future perspective

The clinical potential of measuring serum AMH levels has been evaluated in a wide range of healthcare issues outside assisted reproductive technology, including assessments for ovarian damage caused by chemotherapy, radiation, surgery, and possible predictors for menopause [63, 64].

In the current review, we focused on the usefulness of AMH for evaluating ovarian damage caused by gynecologic interventions, such as surgery and embolization. Regarding endometriomas, 2 randomized studies illustrated that stripping was superior to vaporization or coagulation of the cyst wall in terms of the rates of spontaneous pregnancy and disease recurrence [65–67]. However, vaporization or coagulation has been demonstrated to be more favorable in terms of ovarian reserve [14]. Therefore, the increasingly widespread use of AMH measurements has stimulated research to identify the most appropriate surgical methods for treating endometriomas. As we previously mentioned, several studies revealed that cystectomy for endometriomas causes a decrease of ovarian reserve, especially in cases of bilateral disease. Disturbance of the blood supply has been considered one of the reasons to affect ovarian reserve in endometrioma cystectomy. A small number of studies revealed that suturing or using hemostatic materials without bipolar electrocoagulation might be preferable in the preserving ovarian reserve. Further studies, including those of the rates of pregnancy and recurrence after surgery, are needed to evaluate the true usability of serum AMH levels after endometrioma surgery.

In addition to endometrioma surgery, surgical interventions that might cause ovarian damage should be evaluated in terms of ovarian reserve. It is controversial whether cystectomy for benign ovarian tumors excluding endometriomas negatively affects ovarian reserve. However, the influence should be less than that of cystectomy for endometriomas. UAE is performed for leiomyoma and postpartum hemorrhage. Possible damage to the ovaries is a problem if these patients desire pregnancy in the future. Declines of ovarian reserve possibly caused by salpingectomy, which is occasionally performed for ectopic pregnancy and hydrosalpinx, could also be an issue. Ovarian function is important from the point of view of women’s healthcare. Therefore, considering ovarian function after hysterectomy, the assessment for ovarian reserve using AMH has a role. The studies thus far have not demonstrated a huge influence, although the impact of such interventions on ovarian reserve by is not conclusive.

## Conclusions

In conclusion, serum AMH is helpful for counseling patients who desire future fertility but have benign gynecologic conditions that may require surgical interventions. Serum AMH levels may be preferred in determining the indication and selection of operative methods for benign gynecologic conditions, especially endometriomas. However, there is little available information regarding the correlation between the possibility of live birth and serum AMH levels before and after interventions. Further studies are required to investigate whether and how serum AMH levels in patients with benign gynecologic conditions are relevant to prospective live birth.

## Abbreviations

AMH: Anti-Müllerian hormone
FSH: Follicle-stimulating hormone
IVF: In vitro fertilization
rASRM: Revised american society for reproductive medicine
RCT: Randomized controlled trial
TLH: Total laparoscopic hysterectomy
UAE: Uterine artery embolization.
